# Introducing diabetes emotional well-being into routine consultations for adults with type 2 diabetes

**DOI:** 10.1007/s44357-026-00009-3

**Published:** 2026-08-31

**Authors:** Megan Peck, Iliatha Papachristou Nadal, Vibeke Stenov, Mette Due-Christensen, Sarah Sims, Ruth Harris, Richard I. G. Holt, Jane Speight, Jackie Sturt

**Affiliations:** 1 Florence Nightingale Faculty of Nursing, Midwifery & Palliative Care, King’s College London, London, UK; 2 NAMU - National Center for Applied Research in Older Citizens Living With Multimorbidity, University College Copenhagen, Copenhagen, Denmark; 3 Steno Diabetes Center Copenhagen, Copenhagen, Denmark; 4 Human Development and Health, Faculty of Medicine, University of Southampton, Southampton, UK; 5 School of Psychology, Institute for Health Transformation, Deakin University, Geelong, VIC Australia; 6 The Australian Centre for Behavioural Research in Diabetes, Diabetes Victoria, Melbourne, VIC Australia

**Keywords:** Diabetes distress, Emotional well-being, Rapid realist review, Routine consultation, Type 2 diabetes

## Abstract

**Aims/hypothesis:**

To generate initial programme theories explaining how assessment and discussion of diabetes distress could be embedded into routine type 2 diabetes care, providing a theoretical foundation for future evaluation and implementation.

**Methods:**

A rapid realist review synthesised evidence from qualitative and quantitative studies, systematic reviews and professional commentaries. Four electronic health databases were searched between November 2024 and April 2025, and papers were selected based on their relevance and rigour. Context–mechanism–outcome configurations were developed to hypothesise mechanisms, contextual factors and outcomes associated with integration of diabetes distress assessment and discussion in routine type 2 diabetes care.

**Results:**

36 papers were included in the final synthesis. Five initial programme theories were proposed, which hypothesise that: integration could work when it (1) validates healthcare professionals’ role in providing emotional support; (2) builds skills and confidence to discuss distress; (3) fosters trusting, person-centred relationships and consultations; (4) uses structured tools to facilitate dialogue; and when (5) healthcare professionals normalise routine discussion of emotional well-being. Barriers include time constraints in consultations, workload pressures, limited training and uncertainty regarding professional responsibilities.

**Conclusions/interpretation:**

The review provides a theoretical foundation for understanding how diabetes distress assessment and discussion could work in routine type 2 diabetes care. With future realist evaluation to test and refine these theories, this could address a key gap in existing literature and guide changes in clinical care.

**Graphical Abstract:**

**Figure:**
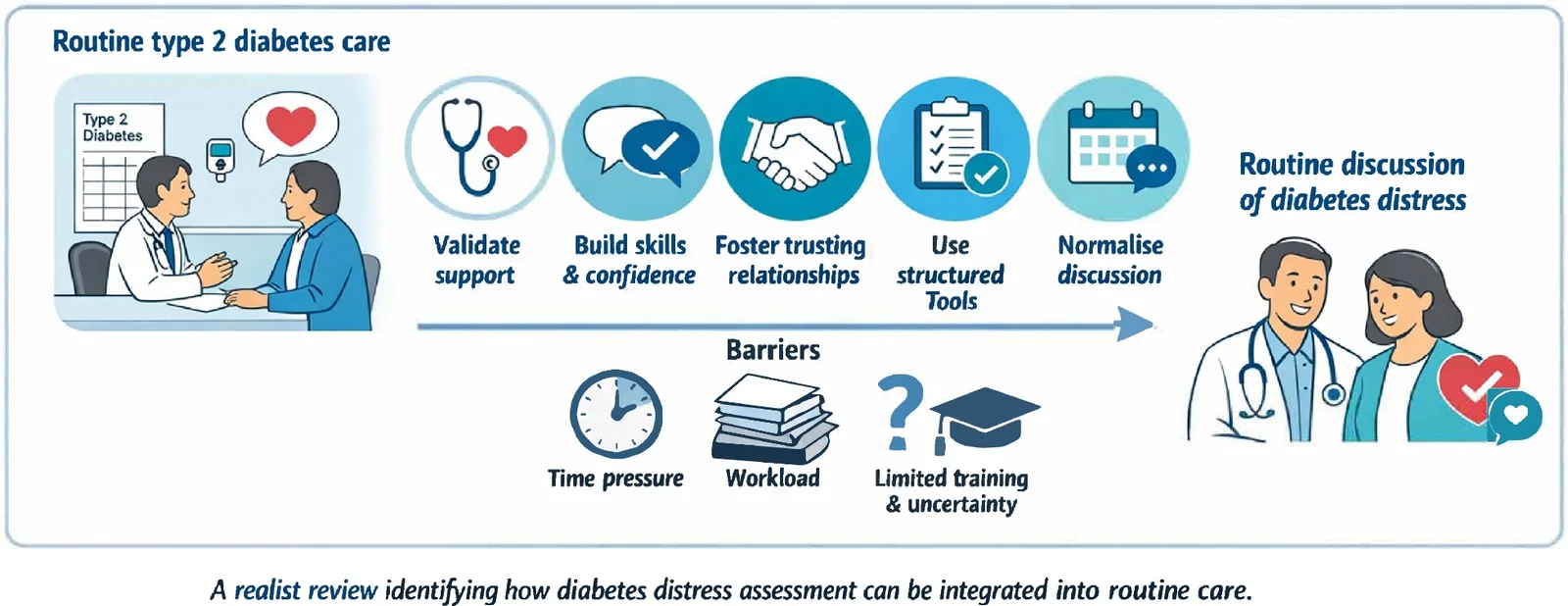

**Supplementary Information:**

The online version contains supplementary material available at https://doi.org/10.1007/s44357-026-00009-3.

**Figure:**
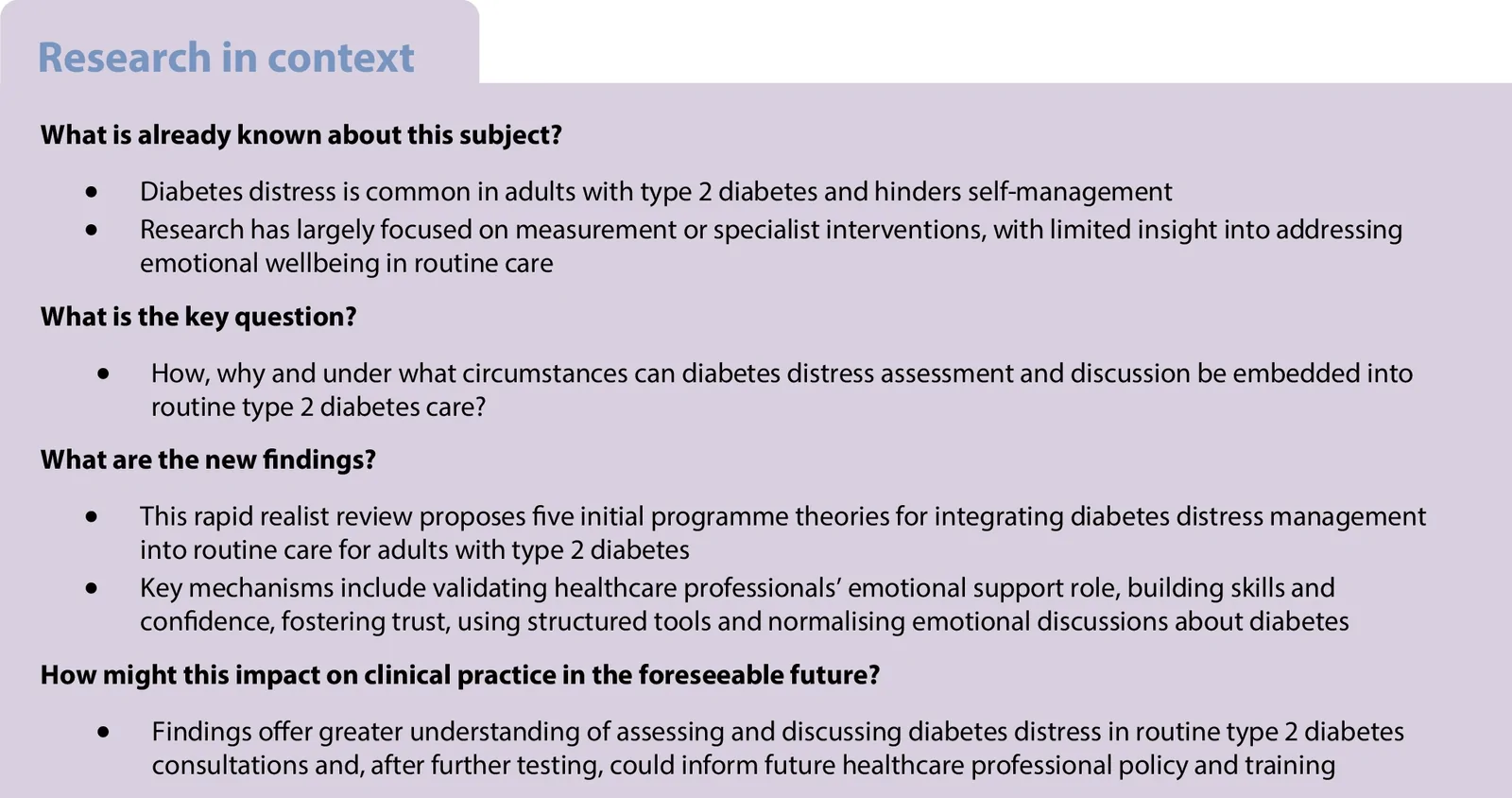

## Introduction

Type 2 diabetes constitutes a growing public health concern globally. According to the International Diabetes Foundation Atlas, 589 million adults are living with diabetes, and the prevalence of diabetes is projected to rise by 46% by 2050 [1]. More than 90% of cases are type 2 diabetes. Achieving glycaemic targets requires sustained self-management across multiple behavioural domains, including taking medication and blood glucose monitoring, while navigating the psychological demands of living with diabetes [2].

People with type 2 diabetes are nearly twice as likely to experience depression and other psychological comorbidities compared with the general population [3]. Diabetes distress, while not a psychiatric condition, is a common emotional response marked by fear, frustration and perceived inadequacy in managing diabetes [4]. Diabetes distress is associated with reduced self-efficacy, impaired self-management, suboptimal glycaemic levels and increased risk of complications [5]. Approximately 36–60% of people with type 2 diabetes experience significantly elevated diabetes distress [6–8].

Despite these implications, diabetes distress remains under-recognised and rarely addressed in routine care. The international second Diabetes Attitudes, Wishes and Needs (DAWN2) study reported that, although 45% of participants experienced diabetes distress, only 24% recalled a healthcare professional asking about the emotional impact of diabetes [9]. National Diabetes UK guidance advocates for consultations to discuss emotional well-being and recommends annual assessment using validated patient-reported outcome measures (PROMs) [10].

PROMs such as the Problem Areas in Diabetes (PAID) scale, the Diabetes Distress Scale and the more recent Type 2 Diabetes Distress Assessment System (T2-DDAS) provide structured methods for identifying, quantifying and understanding diabetes distress [11]. However, their use is shaped by individual needs, healthcare professional attitudes and broader health system factors. These complexities highlight the realist question of ‘what works, for whom, in what contexts and why?’. Understanding how assessments are currently used in consultations is essential for determining how diabetes distress assessment and discussion can be integrated effectively into routine care. This rapid realist review was commissioned as part of the European Society for the Study of Diabetes (EASD) development of its new clinical practice guideline aiming to support the standardisation of the assessment and management of diabetes distress in clinical practice [12]. The aim was therefore to develop initial programme theories theorising how, why and under what circumstances diabetes distress assessment and discussion occur within routine type 2 diabetes care, the contextual factors influencing them and their outcomes for adults with type 2 diabetes, healthcare professionals and healthcare systems.

## Methods

**Fig. 1:**
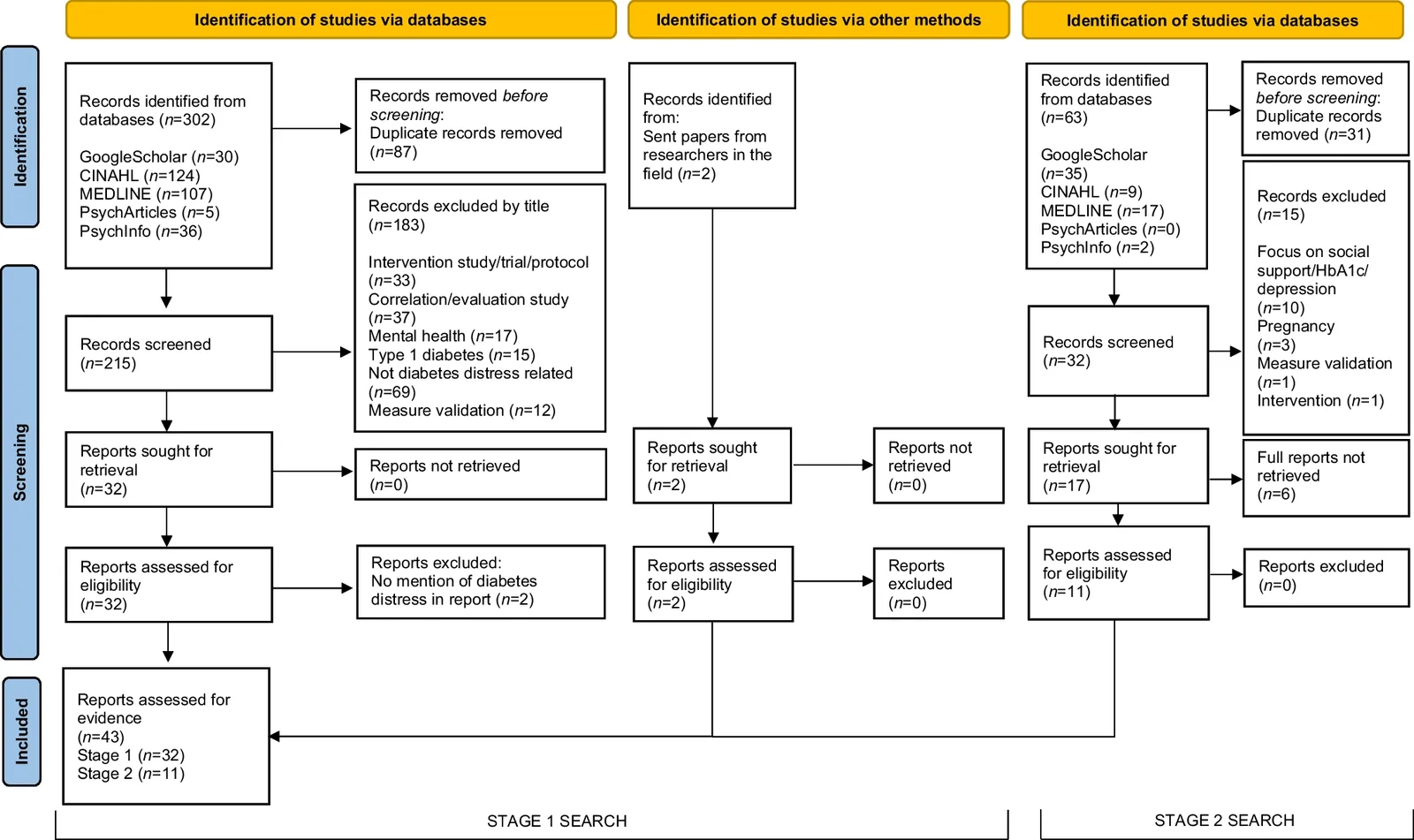
Document flow diagram of the two-stage literature search and study selection process

A rapid realist review was conducted following guidance from Saul et al. (2013) to generate timely evidence while maintaining realist methods [13]. The review focused on diabetes distress in type 2 diabetes to ensure relevance to this specific population. The aim was not exhaustive searching but theoretical saturation, determined by the first author in agreement with the two reviewing members (MD-C and SS) of the authorship team. Realist and Meta-narrative Evidence Synthesis: Evolving Standards (RAMESES) standards [14] were followed (see electronic supplementary material [ESM] 1). Table 1 provides definitions of relevant realist terminology. Figure 1 shows the document flow diagram. Sex and gender were not explicitly incorporated into the review design or synthesis. However, relevant sex and gender-related findings reported in the included studies were noted where available. The review comprised a two-stage literature search and a stakeholder engagement workshop.

**Table 1:** Definitions of realist terms in this rapid realist review

Realist term	Definition
Context (C)	The elements in the background environment that trigger or modify a particular mechanism, which then has an impact on outcomes [15]. Can refer to an individual’s characteristics and capabilities, cultural and societal norms, existing service infrastructures and dynamics, etc
Mechanism (M)	The underpinning agents of action in a programme. A programme offers resources, or opportunities, and how these are taken up depends on the way people respond (their reasoning and active ability to put these into practice) [16]. Mechanisms are context-sensitive, meaning they only get activated in certain contexts
Outcome (O)	The impact or behaviours resulting from the interaction between mechanisms or contexts, intended or unintended
Relevant data (on which CMOCs are based)	Fragments of text that inform understanding contexts, mechanisms and outcomes or the relationship between them. These data may come from results sections (e.g. reported findings), but they may also come from introductions (e.g. theoretical framing), methods (e.g. descriptions of consultation components), discussion sections (e.g. authors’ explanations of why an intervention worked or did not work) or practice guidance documents. What determines inclusion is not the section of the paper, but whether the material contributes to theory building. Sometimes author descriptions and interpretations of data and findings provide relevant data

### Stage 1: literature on the assessment and discussion of diabetes distress in type 2 diabetes care

In stage 1, a search of published literature search was conducted to develop initial programme theories. Between November 2024 and April 2025, four electronic databases were searched (Cumulative Index of Nursing and Allied Health Literature [CINAHL], Medical Literature Analysis and Retrieval System Online [MEDLINE], PsychArticles and PsychInfo) alongside searches of Google Scholar (Advanced Search), using the strategy detailed in ESM 2. Of 302 documents, 32 remained after deduplication.

The 32 documents were read by the first author, and ten were reviewed by two members (MD-C and SS) of the authorship team. A bespoke data extraction form captured information on contexts, outcomes and potential mechanisms. Each reviewer completed the form of extracted relevant data for every document, and discrepancies were resolved through discussion. Reasons for exclusion of documents were recorded. In line with realist methodology, standard quality appraisal (e.g. by study design) was replaced by the first author assessing each document’s ‘fitness for purpose’. This assessment was independently reviewed by two authors (MD-C and SS). The ‘fitness for purpose’ was assessed against two criteria: relevance (whether the document contributed to ongoing theory building) and rigour (whether the method used to generate those data was credible) [14]. This is because, within each document, several pieces of data may contribute to building an initial programme theory, and therefore inclusion assessment cannot be based on the overall study or document quality [14]. Four documents were excluded as they did not add to the synthesis, based on consensus among all three authors (see ESM 3).

When documents were reviewed, the first author used extracted relevant data and retroductive thinking to develop context–mechanism–outcome configurations (CMOCs). Retroductive thinking supported the formulation of initial theories using not just the observable evidence, but also the insight, common sense and informed thinking of the authors [17]. In many cases, mechanisms were not explicitly articulated within the included literature; therefore, retroductive thinking was used to infer plausible generative mechanisms, which involved moving iteratively between the data, author insights and the developing programme theories. CMOC development was iterative, with configurations refined continuously through discussion with the authorship team. This process emphasised the development of theoretical hypotheses rather than definitive explanations, although the CMOC statements are articulated as deterministic statements for testing in future research. Authors contributed contextual expertise in diabetes care and assessment practice, supporting the refinement of mechanisms and ensuring that the emerging theories were grounded in the realities of clinical practice.

Through this process, five overarching initial programme theories and six CMOC statements were developed. Stakeholder involvement also included an online workshop with five adults with type 2 diabetes. This workshop informed minor refinements, including emphasising the relationship between the person with diabetes and the healthcare professional, the importance of healthcare professionals viewing emotional health as part of their role and acknowledging how these conversations may reduce feelings of isolation (see ESM 4 for further detail).

### Stage 2: literature relating to contextual differences between diabetes distress for type 1 diabetes and type 2 diabetes

Between January and April 2025, the stage 2 search sought to improve understanding of any differences in experiences of diabetes distress and to broaden understanding of contextual variation in diabetes distress between type 1 diabetes and type 2 diabetes. The same four electronic databases were searched (CINAHL, MEDLINE, PsychArticles and PsychInfo) alongside Google Scholar (Advanced Search), using the strategy in ESM 5. Of 63 documents, six potentially relevant full-text documents could not be retrieved due to access restrictions, and 11 remained after deduplication. The documents were processed in the same way as those in stage 1: each was assessed for ‘fitness for purpose’ by considering relevance and rigour and relevant data were extracted using the bespoke form to capture information on contexts, outcomes and potential mechanisms. Three documents were excluded as they did not add to the synthesis (see ESM 3). Analysis of the stage 2 literature did not generate additional CMOCs; however, it enabled refinement of the developing programme theories by strengthening understanding of contextual variation between type 1 and type 2 diabetes clinical care. In particular, the literature highlighted differences in care delivery, including the predominance of specialist clinics in type 1 diabetes and primary care management in type 2 diabetes, as well as differences in consultation length, continuity of care and healthcare professional expertise. These contextual distinctions appeared to influence how mechanisms such as relationship building and the use of assessment tools operated in practice. Stage 2 therefore functioned primarily as a theory-testing and contextual refinement phase rather than as a source of new mechanisms.

## Results

The 36 included papers [4, 10, 18, 50] were 25 published research articles, three reviews, four discussion pieces, two measure-development papers, one PowerPoint presentation and one book chapter. Figure 1 presents the document flow, and ESM Table 1 provides brief descriptions. The papers reported work from the USA (*n* = 14), UK (*n* = 5), Australia (*n* = 6), Germany (*n* = 3), the Netherlands (*n* = 3), Iran (*n* = 2), Canada (*n* = 1), Saudi Arabia (*n* = 1) and Japan (*n* = 1), published between 1995 and 2024. The five programme theories and accompanying CMOCs represent theoretical propositions derived through retroductive analysis from the reviewed literature. The CMOC statements are developed as deterministic statements to be tested in future research.

### Initial programme theory 1: extend the scope of diabetes healthcare professionals’ roles to include emotional support

CMOC 1: in clinical contexts in which assessing and discussing diabetes distress is explicitly framed as part of a diabetes healthcare professional’s role, particularly when adults with type 2 diabetes express a preference to discuss diabetes-specific emotions with familiar diabetes healthcare professionals, diabetes healthcare professionals experience a validation of emotional support as within their professional role. This shift in perceived role increases the likelihood that assessment and discussions of diabetes distress are incorporated into routine diabetes care.

Nine documents [20, 26, 27, 29, 31–34, 42] highlighted the importance of the diabetes healthcare professionals’ role in emotional well-being care. This initial theory centres on the existing healthcare practice of prioritising biomedical tasks, with emotional health viewed as secondary.

*27.2% believe(d) that their colleagues think that assessing diabetes distress is an important part of their professional role.*

McMorrow et al. [33]

However, given the high prevalence of diabetes distress, the literature suggests that this prioritisation is unjustified.

*It is hard to define diabetes distress as something ‘extra’ to be viewed outside of day-today diabetes practice.*

Fisher et al. [26]

*There is a need for diabetes clinicians of all stripes to, with adequate support, address diabetes distress so that the emotional side of diabetes becomes just another component of comprehensive diabetes care.*

Fisher et al. [26]

If diabetes healthcare professionals’ views are changed to reflect that emotional management is as important as physical management, this could result in a shift in perception of responsibilities and increased diabetes distress assessment and conversations. Clark et al. [20] reported that, after a diabetes-related distress training session, healthcare professionals reported how diabetes distress assessment had helped them to identify the challenges that people with diabetes were facing, to fulfil what they now viewed as ‘a crucial part of this population’s care’. The team successfully provided comprehensive diabetes distress assessment to all people with type 2 diabetes in their clinic, demonstrating a shared responsibility in upholding and sustaining emotional support [20].

This is important as almost half of adults with type 2 diabetes prefer diabetes-specific emotional discussions with a familiar diabetes professional rather than a mental health specialist who may not understand diabetes.

*47% of those with type 2 diabetes reported wanting to talk with their health professional about their ‘feelings and personal experience of living with diabetes’.*

Hendrieckx et al. [29]

Sharing this expectation could help healthcare professionals to appreciate how emotional support aligns with their role and with what people want from routine care. By receiving emotional support in their routine care, adults with type 2 diabetes are given the opportunity to discuss their emotional well-being with the healthcare professional they know and trust; therefore, their support needs and expectations are met.

*A diabetes healthcare provider was preferred in issues related to diabetes distress. A feeling of trust between the healthcare provider and the person with diabetes was also mentioned as important in stimulating a positive attitude towards psychosocial health care.*

Stoop et al. [42]

### Initial programme theory 2: increase diabetes healthcare professionals’ understanding, skills and confidence to address diabetes distress

CMOC 2: in contexts in which communication techniques, active listening and emotional validation are covered in structured, engaging and evidence-based training, diabetes healthcare professionals develop greater confidence in their skills to discuss diabetes distress. This enhanced sense of capability increases the likelihood that these skills are applied within routine consultations with adults with type 2 diabetes.

CMOC 3: in contexts in which routine diabetes distress assessment frames diabetes distress as a normal emotional response to the demands of living with diabetes rather than a separate mental health condition, healthcare professionals perceive discussions about diabetes distress as less complex and more aligned with their existing skills in practice, increasing their confidence in addressing diabetes distress.

Ten documents addressed this initial programme theory [18, 20, 22, 27, 29, 32, 34, 42]. Many diabetes healthcare professionals already have the well-developed communication skills needed to address diabetes distress, but perceive their skills to be limited when discussing emotional well-being [22]. Training that reinforces existing capabilities and provides practical communication technique training, such as active listening, can increase healthcare professional confidence as these skills are practiced in consultations [18, 22, 27]. By using these skills to engage in more meaningful conversations, adults with type 2 diabetes could subsequently have better consultation experiences if they feel validated and listened to by their healthcare professional.

*A person with diabetes came in and I said to her, 'How are you doing? How are you feeling about everything?’…I realized it was a real can of worms...it was not just about her diabetes. It was about feeling overwhelmed...She actually ended up saying to me, ‘The only reason why this conversation's coming out is because you asked me how I am. Had you not asked me, I wouldn't tell you…’*

Halliday et al. [27]

However, without sufficient training and support, many diabetes healthcare professionals do not feel they have the required skills, knowledge and experience to adequately provide emotional well-being care within their consultations. Many report limited training; in one clinic, 92% had never received training on diabetes distress [20].

*‘I have no experience (with diabetes distress assessments) so they aren’t part of my usual routine in diabetes management.’*

McMorrow et al. [33]

*Healthcare providers mentioned that factors that impeded addressing psychosocial issues by the diabetes healthcare provider were …not feeling sufficiently competent in addressing these issues.*

Stoop et al. [42]

Even with improved healthcare professional confidence and skills, time constraints will often remain a barrier, especially concerns about ‘opening a can of worms’ in a short consultation period. This reinforces the value of reframing diabetes distress as part of routine practice rather than an additional task.

### Initial programme theory 3: foster a safe and collaborative relationship through person-centred, attentive interactions

CMOC 4: in contexts in which consultations are routinely facilitated in a collaborative, person-centred manner, characterised by empathy, compassion and sufficient time, adults with type 2 diabetes feel heard and respected, and they can be expected to feel psychologically safe to disclose emotional concerns. This enables diabetes healthcare professionals to gain a better understanding of their needs and perspectives, increasing the likelihood that care is experienced as more holistic and collaborative.

Eight documents [19, 28, 29, 32, 36, 37, 39, 42] addressed the shift from a directive, hierarchical consultation style to a collaborative, person-centred approach. Rather than dominating discussions and delivering information and solutions, diabetes healthcare professionals can listen attentively and work as partners with adults with type 2 diabetes, offering understanding and support of their experiences.

*Patient-centred Decision-making and Compassionate/Respectful were significantly associated with reduced diabetes distress.*

Peimani et al. [36]

In doing this, adults with type 2 diabetes can feel safe, heard and respected, and they can be expected to feel more comfortable within their consultation to disclose how they are feeling. This supports a more equal, collaborative dialogue, which is associated with reduced diabetes distress. This also helps diabetes healthcare professionals to better understand individual experiences and needs.

*By listening to patients’ struggles, providers can better understand the sources behind their diabetes distress and more effectively troubleshoot patient-centred ways to support them in reducing that distress.*

Buys et al. [19]

### Initial programme theory 4: provide a valid and reliable diabetes distress assessment tool that prompts reflection and articulation of feelings for adults with type 2 diabetes

CMOC 5: in contexts in which adults with type 2 diabetes are introduced to a valid and reliable diabetes distress assessment tool that captures a range of negative emotional responses to diabetes, the tool prompts reflection and helps individuals to recognise and articulate how diabetes affects their everyday life. This process increases understanding for both adults with type 2 diabetes and their healthcare professional of how diabetes distress impacts them and supports more meaningful discussions about emotional concerns within consultations.

Twelve documents addressed this initial programme theory [4, 8, 11, 21, 23, 24, 29–31, 33, 38, 40], demonstrating the importance attributed to a valid assessment tool and its multiple functions for both diabetes healthcare professionals and adults with type 2 diabetes. These tools capture the range of negative emotional responses to living with type 2 diabetes and can act as topic guides for discussions in consultations. Emotional reactions to diabetes are not always routinely reflected on by adults with type 2 diabetes; assessment can prompt this recognition. Using an assessment tool can support them in expressing their emotions by using the language of the assessment to mirror and articulate their emotional responses.

*Measures elicit an emotional reaction to, rather than cognitive reaction on, diabetes (i.e. items elicit how they feel rather than how they think about diabetes).*

Dennick et al. [21]

This can drive consultations that are more inclusive of the whole emotional experience of living with diabetes. By understanding the emotional side of diabetes, healthcare professionals have the opportunity to gain a greater understanding of how this impacts ability, motivation and confidence to carry out self-management behaviours.

*Based on our findings, inviting people with diabetes to complete a measure of diabetes distress…may facilitate the conversation about their needs and concerns…As part of a person-centred communication, has been shown to reduce unmet needs, improve a person’s overall satisfaction with the interactions with their health professional and increase the health professional’s understanding of their problems, without increasing consultation time.*

Hendrieckx et al. [29]

### Initial programme theory 5: ensure regular assessment and discussion of diabetes distress

CMOC 6: in contexts in which diabetes healthcare professionals routinely assess and discuss diabetes distress during regular appointments, adults with type 2 diabetes perceive emotional reactions to diabetes as common and normal experiences that are important to talk about. This reduces feelings of isolation and stigma as emotional concerns are discussed alongside physical health in routine care.

Ten documents addressed this initial programme theory [10, 11, 18, 20, 25, 29, 32, 33, 41], which highlights how the regular assessment and discussion of emotional well-being in consultations can give adults with type 2 diabetes the regular space to verbalise their feelings and normalise their experiences. People with lived experience of type 2 diabetes suggest that having these conversations in routine care could help individuals experiencing distress to feel less alone and less isolated. They also believe that it may help to reduce the stigma associated with speaking openly about their feelings. Given that diabetes distress fluctuates over time, routine diabetes distress conversations can ensure that no one experiencing elevated diabetes distress is left feeling unsupported at any given consultation.

*Our findings suggest that the assessment of distress-related disorders in patients with diabetes at successive patient visits provides a more clinically relevant estimate of their prevalence than when they are assessed only at one point in time.*

Fisher et al. [25]

*As diabetes distress is relatively common, and also impacts upon self-care, it is important that every consultation includes opportunity for the person to express how they are feeling about life with diabetes.*

Diabetes UK [10]

## Discussion

This rapid realist review identified five initial programme theories explaining how the assessment and discussion of diabetes distress could improve the patient experience of routine type 2 diabetes care. The initial programme theories suggest that routine assessment, discussion and follow-up of diabetes distress creates a space for adults with type 2 diabetes to feel recognised, to reflect and articulate emotional issues, to feel less alone and accepting of emotional reactions and to feel that their concerns are normal. Using structured tools can facilitate reflection and dialogue from adults with type 2 diabetes, and embedding regular assessment and discussion of diabetes distress can normalise attention to emotional well-being within diabetes consultations. These mechanisms emphasise reciprocal benefits: adults with type 2 diabetes feel supported and heard, while healthcare professionals fulfil a crucial missing part of diabetes care. At the same time, barriers such as limited consultation time, workload pressures and uncertainty about professional responsibilities remain important contextual challenges.

The review offers a theoretical contribution by articulating initial programme theories that hypothesise why and under what circumstances integrating emotional well-being support can improve routine clinical consultations. The findings highlight that attention to diabetes distress should not be viewed as an optional task or the remit of mental health specialists alone. Instead, it should be considered an integral component of person-centred diabetes care. The first two initial programme theories point to the importance of professional training, while the remaining theories illustrate how consultation dynamics, structured assessment tools and the normalisation of diabetes distress discussions can make emotional care part of routine practice.

The findings are consistent with previous reviews and empirical studies that emphasise the importance of recognising and addressing diabetes distress in clinical settings [32, 34]. Prior research has typically focused on either the measurement of diabetes distress [21, 31] or the effectiveness of psychosocial interventions [26]. This review extends the evidence base by theorising how diabetes distress assessment and discussion can work within standard care pathways. Earlier studies have often expressed scepticism about whether emotional well-being can be meaningfully discussed within the constraints of brief consultations [51]. This review suggests that reframing diabetes distress as a normal part of living with diabetes, and using validated tools to structure discussion, may help to overcome these concerns.

In contrast to much of the existing literature, which often considers populations with type 1 diabetes and type 2 diabetes together, this review focuses specifically on the context of type 2 diabetes. A separate realist review was conducted focused on adults with type 1 diabetes, using different literature searching methodology due to differing purposes and commissioning [52]. Comparison with the initial programme theories developed for adults with type 1 diabetes indicates broad similarities in mechanisms, including the importance of trust, normalisation of emotional burden and use of structured tools to facilitate discussion. However, contextual factors appear to influence how these mechanisms operate in practice. Type 1 diabetes care is often delivered in specialist clinics with longer, more frequent appointments, which may support more in-depth conversations and skill development for healthcare professionals. By contrast, type 2 diabetes is frequently managed in primary care, where consultations are shorter and clinicians may have less specialist support, requiring adaptations in the use of structured tools and strategies to build trust efficiently. These contextual differences suggest that, although the underlying mechanisms are similar, their expression and implementation may need to be tailored to the care setting and patient population.

A key strength of this review is its use of realist methodology, which enabled theory-driven synthesis and provided insight into mechanisms and contextual factors that conventional effectiveness reviews cannot capture. The breadth of included materials, including empirical studies, systematic reviews and commentaries, generated a greater understanding of how and why emotional well-being care could be integrated into routine practice, particularly given the limited testing of effectiveness in the included literature. Stakeholder engagement further strengthened the review, enhancing theory refinement through the sharing of lived experience.

However, several limitations should be acknowledged. Many of the included papers lacked sufficient detail to fully articulate CMOCs, which limited the ability to develop comprehensive programme theories and required greater reliance on authors’ retroductive thinking to produce hypotheses to be tested. There are also several limitations of the search strategy. Although five major databases were searched, relevant studies may still have been missed, particularly grey literature sources. We acknowledge that we did not aim for exhaustive searching. Screening and selection of studies were undertaken by a single author, which may have increased the risk of selection bias. Sex and gender-based analysis was not undertaken because reporting of sex and gender within the included studies was inconsistent. Consequently, this review could not assess whether contexts, mechanisms or outcomes varied by sex or gender.

The findings carry important implications for further research that could inform future policy and practice. The initial programme theories generated provide an explanatory framework that will guide future testing. For example, they suggest that attention to emotional well-being might be more effectively integrated into diabetes care when supported by structured training, organisational endorsement and the use of validated assessment tools. A realist evaluation is planned to explore whether and how assessment and discussion of diabetes distress can be embedded effectively into routine type 2 diabetes care, testing and refining the programme theories developed in this review.

## Conclusion

In summary, this rapid realist review has generated initial programme theories that provide a theoretical foundation to understand how the assessment, discussion and follow-up of diabetes distress can work in routine type 2 diabetes consultations. With the future realist evaluation aiming to test and refine these theories in practice, these insights could address the gap identified in existing literature by theorising how routine practice can accommodate emotional support for adults with type 2 diabetes. This could ensure that attention to emotional well-being becomes central to, rather than separate from, diabetes care.

## Supplementary Information

Below is the link to the electronic supplementary material.

Supplementary file1 (PDF 598 KB)

## Data Availability

The present study is a review and does not involve the generation or analysis of new data. All data and materials referenced in this article are derived from previously published documents that are publicly available through the original sources.

## Abbreviations

CMOC: Context–mechanism–outcome configuration
PROM: Patient-reported outcome measure

## Code availability

This study did not generate or use any custom code. All analyses were qualitative and based on previously published documents.
