# Supplementary material for: Introducing diabetes emotional well-being into routine consultations for adults with type 2 diabetes
Source: Metabologia. 2026 Aug 31;2(1):9. doi: 10.1007/s44357-026-00009-3 (PMC13529831; doi:10.1007/s44357-026-00009-3)
Supplement: Supplementary file 1 [file 44357_2026_9_MOESM1_ESM.pdf]

Electronic supplementary material (ESM)

ESM 1 – RAMESES reporting guidelines

TITLE	
1	In the title, identify the document as a realist synthesis or review
ABSTRACT	
2	While acknowledging publication requirements and house style, abstracts should ideally contain brief details of the study's background, review question or objectives; search strategy; methods of selection, appraisal, analysis and synthesis of sources; main results; and implications for practice.
INTRODUCTION	
3 Rationale for review	Explain why the review is needed and what it is likely to contribute to existing understanding of the topic area.
4 Objectives and focus of review	State the objective(s) of the review and/or the review question(s). Define and provide a rationale for the focus of the review.
METHODS	
5 Changes in review process	Any changes made to the review process that was initially planned should be briefly described and justified.
6 Rationale for using realist review	Explain why realist synthesis was considered the most appropriate method to use.
7 Scoping the literature	Describe and justify the initial process of exploratory scoping of the literature.
8 Searching processes	While considering specific requirements of the journal or other publication outlet, state and provide a rationale for how the iterative searching was done. Provide details on all the sources accessed for information in the review. Where searching in electronic databases has taken place, the details should include, for example, name of database, search terms, dates of coverage and date last searched. If individuals familiar with the relevant literature and/or topic area were contacted, indicate how they were identified and selected.

9 Selection and appraisal of documents	Explain how judgements were made about including and excluding data from documents and justify these.
10 Data extraction	Describe and explain which data or information were extracted from the included documents and justify this selection
11 Analysis and synthesis processes	Describe the analysis and synthesis processes in detail. This section should include information on the constructs analysed and describe the analytic process.
RESULTS	
12 Document flow diagram	Provide details on the number of documents assessed for eligibility and included in the review with reasons for exclusion at each stage as well as an indication of their source of origin (for example, from searching databases, reference lists and so on).
13 Document characteristics	Provide information on the characteristics of the documents included in the review.
14 Main findings	Present the key findings with a specific focus on theory building and testing.
DISCUSSION	
15 Summary of findings	Summarize the main findings, taking into account the review's objective(s), research question(s), focus and intended audience(s).
16 Strengths, limitations and future research directions	Discuss both the strengths of the review and its limitations. These should include (but need not be restricted to) (a) consideration of all the steps in the review process and (b) comment on the overall strength of evidence supporting the explanatory insights which emerged. The limitations identified may point to areas where further work is needed.
17 Comparison with existing literature	Where applicable, compare and contrast the review's findings with the existing literature (for example, other reviews) on the same topic.
18 Conclusions and recommendations	List the main implications of the findings and place these in the context of other relevant literature. If appropriate, ofer

	recommendations for policy and practice.
19 Funding	Provide details of funding source (if any) for the review, the role played by the funder (if any) and any conflicts of interests of the reviewers.

ESM 2 – Stage 1 search strategy

Terms and operators

“type 2 diabetes” OR “T2D” OR “diabetes type 2”

AND

“diabetes-related distress” OR “diabetes distress” OR “diabetes specific emotional distress”

AND

“assessment” “assessing “tool” “measure” “scale” “questionnaire”

NOT

“Intervention”

[**Limiters:** terms in Title, English-language only]

Separate search

“type 2 diabetes” OR “T2D” OR “diabetes type 2”

AND

“diabetes-related distress” OR “diabetes distress” OR “diabetes specific emotional distress”

AND

“conversation” OR “consultation” OR “discussion”

[**Limiters:** terms in Title, English-language only]

ESM 3 – Excluded documents in the review process

Stage 1

Hirsch A, Bartholomae C, Volmer T. Dimensions of quality of life in people with non-insulin-dependent diabetes. Qual Life Res. 2000;9(2):207-18. https://doi.org/10.1023/A:1008959810698

Gonzalez JS, Delahanty LM, Safren SA, Meigs JB, Grant RW. Diferentiating symptoms of depression from diabetes-specific distress: relationships with self-care in type 2 diabetes. Diabetologia. 2008;51(10):1822-5. doi:10.1007/s00125-008-1113-x.

Perrin NE, Davies MJ, Robertson N, Snoek FJ, Khunti K. The prevalence of diabetes-specific emotional distress in people with type 2 diabetes: a systematic review and meta-analysis. Diabet Med. 2017;34(11):1508-20. https://doi.org/10.1111/dme.13448

Freeman-Hildreth Y, Aron D, Cola PA, Wang Y. Coping with diabetes: provider attributes that influence type 2 diabetes adherence. PLoS One. 2019;14(4):e0214713. https://doi.org/10.1371/journal.pone.0214713

Stage 2

Mach C, Bulanadi J, Gucciardi E, Segal P, De Melo M. Exploring the needs of adults living with type 1 or type 2 diabetes distress using the Problem Areas in Diabetes 5 Tool. Can J Diabetes. 2023;47(1):51-57.e1. https://doi.org/10.1016/j.jcjd.2022.07.008

Nhlabatsi ML, van Teijlingen ER, Hundley VA. Clinicians' barriers to screening and diagnosing diabetes distress in patients with type 1 and 2 diabetes mellitus: a systematic review. Afr J Health Sci. 2023;36(5):Art. 5. https://doi.org/10.4314/ajhs.v36i5.2

Zara S, Kruse J, Leveling AM, Schmitz J, Hempler I, Kampling H. Capturing diabetes-related distress and burden from the perspective of patients with type 1 or type 2 diabetes: protocol for an explorative mixed methods study. JMIR Res Protoc. 2022;11(8):e38477. https://doi.org/10.2196/38477

ESM 4 - Detail of stakeholder involvement

In addition to the authorship team's expertise, stakeholder involvement also included an online workshop with five adults with type 2 diabetes. We organised an online workshop with five adults with type 2 diabetes to discuss our emerging theories and gain feedback. The workshop's goal was to refine our understanding of how emotional distress could be addressed within routine care, ensuring that our theories accurately reflected the lived experiences of people with diabetes and the practical realities of clinical settings.

Before the workshop, we shared the key elements of our work so far - our initial overarching programme theories, broadly summarising the CMOC statements we hypothesised. We made the materials accessible to everyone in lay language, recognising that not all would be familiar with the language of academic research and healthcare jargon. We also sent out clear instructions about the workshop process and encouraged participants to think about their personal experiences with diabetes distress or emotional wellbeing in their diabetes teams.

We started the workshop by presenting the initial programme theories and the if-then-because statements. The theories seemed to immediately resonate with the group. For example, the idea that emotional support during consultations could lead to greater engagement in self-management was broadly agreed upon. One participant shared a story about a time when emotional support from a healthcare provider had helped them feel listened to. Another participant emphasised how they often felt ignored or dismissed as a person, as if all that mattered were their diabetes numbers, which made taking to their healthcare professional dificult for them.

As we explored the CMOC statements, it became clear how much the relationship between the person with diabetes and the healthcare professional shaped their experience of diabetes care. The participants shared stories of both positive and negative interactions with healthcare professionals. One key insight that emerged was the importance of trust. Participants agreed with each other that without trust in their healthcare provider, emotional distress couldn't be efectively addressed.

One takeaway was the need to emphasise that emotional health should be considered a part of a diabetes healthcare professional's role. Several participants expressed that they felt their diabetes healthcare professionals often overlooked the emotional aspect of living with a chronic condition like diabetes and the complications it can cause. They suggested that incorporating discussions of emotional wellbeing into routine care, rather than treating it as an afterthought, would be better for them. They also talked about the value of practical tools for guiding conversations about emotional distress. These tools could serve as an important mechanism to ensure that discussions of emotional wellbeing have a set way of being discussed, so the person with diabetes

knows what is going to be discussed and the healthcare professional can tailor discussions to areas that are needed. It was also suggested that by having these conversations in clinic, there would be less stigma around talking about feelings, and people wouldn't feel like they were the only ones experiencing distress relating to diabetes.

We suggested revisions in the relevant if-then-because statements, and the group agreed that these were reflective of their feedback.

ESM 5 - Stage 2 search strategy

Terms and operators
“type 2 diabetes” OR “T2D” OR “diabetes type 2”
AND
“type 1 diabetes” OR “T1D” OR “diabetes type 1”
AND
“diabetes-related distress” OR “diabetes distress” OR “diabetes specific emotional distress”
[**Limiters:** terms in Title, English-language only]

ESM Table 1 - Thirty-six papers included in the review

Reference Number	Author	Date	Title	Type of document	Country of publication	Database source
16	Balini et al.	2024	Diabetes-related Distress in People with Type 2 Diabetes: Experience from a Community Diabetes Clinic	Research article	UK	Identified via database search CINAHL
17	Buys et al.	2024	Motivational Interviewing: A Tool for Managing Diabetes Distress	Discussion paper	US	Identified via database search GoogleScholar
18	Clark et al.	2024	Diabetes Self-Management Support through Diabetes-related Distress Awareness	Research article	US	Identified via database search CINAHL
19	Dennick, Sturt & Speight	2017	What is diabetes distress and how can we measure it? A narrative review and conceptual model	Narrative review	UK	Identified via database search CINAHL
20	Diabetes UK	2019	The Diabetes and Emotional Health Handbook Chapter 3: Diabetes Distress	Online book chapter	UK	Sent from researcher on D-stress team
21	Fenwick et al.	2018	What is the best measure for assessing diabetes distress? A comparison of the Problem Areas in Diabetes and	Discussion paper	Australia	Identified via database search CINAHL

			Diabetes Distress Scale: results from Diabetes MILES-Australia			
22	Fisher et al.	2007	Clinical depression versus distress among patients with type 2 diabetes	Research article	US	Identified via database search CINAHL & MEDLINE
23	Fisher et al.	2008	A longitudinal study of afective and anxiety disorders, depressive afect and diabetes distress in adults with type 2 diabetes	Research article	US	Identified via database search CINAHL
8	Fisher et al.	2022	A new perspective on diabetes distress using the type 2 diabetes distress assessment system (T2-DDAS): Prevalence and Change over time	Research article	US	Identified via database search CINAHL & MEDLINE
24	Fisher et al.	2024	Bringing the Assessment and Treatment of Diabetes Distress into the Real World of Clinical Care: Time for a Shift in Perspective	Discussion paper	US	Identified via database search CINAHL & MEDLINE
25	Halliday et al.	2020	The Diabetes and Emotional Health	Research article	Australia	Identified via database search

			Handbook and Toolkit for Health Professionals Supporting Adults With Type 1 Diabetes and Type 2 Diabetes: Formative Evaluation			MEDLINE
26	Halliday et al.	2024	An eLearning to Upskill Health Professionals in Providing Support for Diabetes Distress	PowerPoint Presentation	Australia	Sent from researcher on D-stress team
27	Hendrieckx et al.	2021	Adults with Diabetes Distress often want to talk with their Health Professionals about it: Findings from an Audit of 4 Australian Specialist Diabetes Clinics	Research article	Australia	Identified via database search GoogleScholar
28	Herschbach et al.	1997	Psychometric Properties of the Questionnaire on Stress in Patients with Diabetes – Revised (QSD-R)	Research article	Germany	Identified via database search MEDLINE
29	Hood et al.	2018	Understanding Diabetes-Related Distress Characteristics and Psychosocial Support Preferences of Urban African American Adults	Research article	US	Identified via database search CINAHL & MEDLINE

			living with Type 2 Diabetes			
30	McGrath et al.	2021	Barriers and Enablers to Screening and Diagnosing Diabetes Distress and Depression in People with Type 2 Diabetes Mellitus: A qualitative Evidence Synthesis	Review	UK	Identified via database search CINAHL & MEDLINE
31	McMorrow et al.	2022a	Assessing and addressing diabetes distress among adults with type 2 diabetes: An online survey of Australian general practitioners	Research article	Australia	Identified via database search CINAHL & MEDLINE
32	McMorrow et al.	2022b	Efect of routinely assessing and addressing depression and diabetes distress on clinical outcomes about adults with type 2 diabetes: a systematic review	Systematic review	Australia	Identified via database search PsychInfo
33	Parsa et al.	2019	Diabetes distress and its clinical determinants in patients with type 2 diabetes	Research article	Iran	Identified via database search MEDLINE
34	Peimani et al.	2022	Type 2 Diabetes Burden and Diabetes	Research article	Iran	Identified via database search

			Distress: The Bufering Efect and Patient-centred Communication			CINAHL & MEDLINE
11	Polonksy et al.	2022	Toward a more comprehensive understanding of the emotional side of type 2 diabetes: A re-envisioning of the assessment of diabetes distress	Research article	US	Identified via database search MEDLINE
35	Polonsky et al.	2017	Physician-Patient Communication at Diagnosis of Type 2 Diabetes and Its Links to Patient Outcomes: New Results from the Global IntroDia Study	Research article	US	Identified via database search CINAHL & MEDLINE
36	Polonsky et al.	1995	Assessment of Diabetes-related Distress	Scale development	US	Identified via database search CINAHL
37	Polonsky et al.	2005	Assessing Psychosocial Distress in Diabetes: Development of the Diabetes Distress Scale	Scale development	US	Identified via database search CINAHL & MEDLINE
38	Rutten et al.	2020	Person-centred Diabetes Care and Patient Activation in People with Type 2 Diabetes	Research article	Netherlands	Identified via database search GoogleScholar
39	Schmitt et al.	2016	How to assess diabetes distress: Comparison of	Research article	Germany	Identified via database search CINAHL &

			the Problem Areas in Diabetes Scale (PAID) and the Diabetes Distress Scale (DDS)			MEDLINE
40	Speight et al.	2012	Generic and diabetes-specific well-being in the AT.LANTUS Follow-on study: Further psychometric validation of the W-BQ28 indicates its utility in research and clinical practice in Type 2 diabetes in the UK	Research article	UK	Identified via database search CINAHL & MEDLINE
41	Stoop et al.	2019	Psychosocial healthcare needs of People with Type 2 Diabetes in Primary Care: Views of Patients and Healthcare Providers	Research article	Netherlands	Identified via database search CINAHL
42	Coccaro et al.	2020	Emotional Regulation and Diabetes Distress in Adults with Type 1 and Type 2 Diabetes	Research article	US	Identified via database search CINAHL & MEDLINE
43	de Groot et al.	2023	The Diabetes Tune-Up Group: A Multidisciplinary Approach to Improve	Research article	US	Identified via database search MEDLINE

			Diabetes Distress and A1C Among Adults with Type 1 and Type 2 Diabetes			
44	DeMolitor et al.	2020	Diabetes distress in Adults Living with Type 1 and Type 2 Diabetes: A public health issue	Discussion paper	Canada	Identified via database search CINAHL & MEDLINE
45	Embaye et al.	2024	Coping Strategies for Managing Diabetes Distress in Adults with Type 1 and Type 2 Diabetes: A cross-sectional Study on Use and Perceived Usefulness	Research article	Netherlands	Identified via database search MEDLINE
46	Kuniss et al.	2019	Diabetes-related Distress is Reduced in People with Type 1 Diabetes, but not Type 2 Diabetes, after Participating in a Diabetes Treatment and Teaching Programme	Research article	Germany	Identified via database search GoogleScholar
47	Miyawaki et al.	2015	Diferences in Emotional Distress among Inpatients with Type 1, Obese Type 2, and Non-Obese Type 2 Diabetes Mellitus	Research article	Japan	Identified via database search MEDLINE

48	Moawd	2022	Quality of Life in University Students with Diabetes Distress: Type 1 and Type 2 Diabetes Diferences	Research article	Saudi Arabia	Identified via database search MEDLINE
49	Orben et al.	2022	Diferences and Similarities in the Experience of Living with Diabetes Distress: A Qualitative Study of Adults with Type 1 and Type 2 Diabetes	Research article	US	Identified via database search CINAHL & MEDLINE
